# In Vitro and In Vivo Studies of a Verapamil-Containing Gastroretentive Solid Foam Capsule

**DOI:** 10.3390/pharmaceutics14020350

**Published:** 2022-02-02

**Authors:** Ádám Haimhoffer, Gábor Vasvári, István Budai, Monika Béresová, Ádám Deák, Norbert Németh, Judit Váradi, Dávid Sinka, Ildikó Bácskay, Miklós Vecsernyés, Ferenc Fenyvesi

**Affiliations:** 1 Department of Pharmaceutical Technology, Faculty of Pharmacy, University of Debrecen, Nagyerdei St. 98, H-4032 Debrecen, Hungary; haimhoffer.adam@pharm.unideb.hu (Á.H.); vasvari.gabor@pharm.unideb.hu (G.V.); varadi.judit@pharm.unideb.hu (J.V.); sinka.david@pharm.unideb.hu (D.S.); bacskay.ildiko@pharm.unideb.hu (I.B.); vecsernyes.miklos@pharm.unideb.hu (M.V.); 2 Doctoral School of Pharmaceutical Sciences, University of Debrecen, H-4032 Debrecen, Hungary; 3 Faculty of Engineering, University of Debrecen, Ótemető Street 2-4, H-4028 Debrecen, Hungary; budai.istvan@eng.unideb.hu; 4 Department of Medical Imaging, Faculty of Medicine, University of Debrecen, Nagyerdei krt. 94, H-4032 Debrecen, Hungary; beres.monika@med.unideb.hu; 5 Department of Operative Techniques and Surgical Research, Faculty of Medicine, University of Debrecen, Móricz Zsigmond u. 22, H-4032 Debrecen, Hungary; deak.adam@med.unideb.hu (Á.D.); nemeth@med.unideb.hu (N.N.); 6 Institute of Healthcare Industry, University of Debrecen, Nagyerdei St. 98, H-4032 Debrecen, Hungary

**Keywords:** solid foam, gastric retention, verapamil, continuous production, pharmacokinetic study

## Abstract

Gastroretentive systems may overcome problems associated with incomplete drug absorption by localized release of the API in the stomach. Low-density drug delivery systems can float in the gastric content and improve the bioavailability of small molecules. The current publication presents verapamil–HCl-containing solid foam prepared by continuous manufacturing. Production runs were validated, and the foam structure was characterized by micro-CT scans and SEM. Dissolution properties, texture changes during dissolution, and floating forces were analyzed. An optimized formulation was chosen and given orally to Beagle dogs to determine the pharmacokinetic parameters of the solid foam capsules. As a result, a 12.5 m/m% stearic acid content was found to be the most effective to reduce the apparent density of capsules. Drug release can be described by the first-order model, where 70% of verapamil dissolved after 10 h from the optimized formulation. The texture analysis proved that the structures of the solid foams are resistant. Additionally, the floating forces of the samples remained constant during their dissolution in acidic media. An in vivo study confirmed the prolonged release of the API, and gastroscopic images verified the retention of the capsule in the stomach.

## 1. Introduction

Nowadays, one of the most important challenges in pharmaceutical technology is the development of drug delivery systems (DDS). There are five generations of DDS. The first and second generations include conventional therapeutic drug carriers such as tablets, capsules, granules, or their enteric coating versions. The group of controlled DDS is the third generation, which is now a focus due to the reduced dosing frequency or enhanced drug efficacy compared with conventional formulations [1]. In addition, the number of newly synthesized active ingredients has decreased in recent decades, and the emphasis has been on converting existing active ingredients into renewed dosage forms. Drug delivery systems provide a new opportunity to increase the usability of current drugs in certain diseases or to improve patient compliance.

Gastroretentive therapeutic systems are one of the major groups of prolonged or controlled release systems [2]. The success of gastroretentive therapy has been described in relation to a number of active pharmaceutic ingredients (APIs), including, but not limited to, the following: some antibiotics (amoxicillin, levofloxacin, ciprofloxacin, metronidazole [2,3]), antiviral agents (acyclovir, zidovudine, lamivudine [4,5,6]), antihypertensive drugs (carvedilol, prazosin, verapamil [7,8,9]), and some other drugs (levodopa, drotaverine, famotidine, metformin [10,11,12,13]).

Three relevant technologies are known for their use in gastroretentive (GR) formulations. The first technology aids in the binding of excipients or the dosage form to the mucin layer that largely covers the stomach surface; these are called mucoadhesive GR systems. These systems aid in the adhesion of biopolymers to the stomach surface and release the API in a prolonged manner [14,15,16]. The expanding devices are a new development, and they prevent transmission through the pyloric sphincter through a size increase caused by the interaction with gastric juice [17]. Density-based systems have been used to increase the bioavailability of drugs by gastroretention since 1986 [2,18]. These systems can float on the surface of stomach juice or sink to the bottom of the stomach to avoid passage into the duodenum [19,20,21]. High-density formulations are more resistant to stomach motion and transport, so they anchor at the bottom of the stomach and release the API [22]. Low-density formulations have been shown to remain in the stomach longer than other systems which have a similar density to gastric fluid [18,23].

Recently, a new technology was developed by our group to prepare foam from hot and molten dispersions at atmospheric pressure. This process is based on the dispersion of air into a molten suspension to create a foam that has a hard structure after cooling down at room temperature. The developed foaming device is suitable for the continuous production of low-density, molded, solid dosage forms that can float immediately. Due to the abovementioned properties, the foamed dosage form is a GR system, and its formation has been confirmed by in vitro and in vivo tests [3,24]. To date, we have not prepared an API-containing formulation through continuous production with the foaming device that has been subjected to in vivo pharmacokinetic testing. In this study, we researched the formulation and absorption of verapamil and investigated the pharmacokinetic properties of the formed solid foam for the first time.

Our aim was to increase the bioavailability of a systemically active substance, verapamil–HCl, with a GR DDS that was produced with the abovementioned technology. Verapamil belongs to the first class of the Biopharmaceutics Classification System (BCS), having high solubility and high permeability [25]. It is well absorbed from the gastrointestinal tract, while it has low bioavailability (<20%) due to its lower solubility in the higher pH of the intestine (0.44 mg/mL at pH = 7.32) and short half-life (t1/2 is approximately 5 h) [7,26,27]. According to earlier results, gastroretention can improve the bioavailability of verapamil [9,22]. All types of GR systems are used in verapamil formulations, including low-density [19,20,21], high-density [22], and mucoadhesive systems [14,15,16]. Nevertheless, to the best of our knowledge, the active substance has not yet been marketed in a gastrointestinal formulation in Europe. Our aim was to develop a successful and marketable formulation with our already-published method by eliminating the shortcomings of the published formulations that have already been described.

## 2. Materials and Methods

### 2.1. Materials and Experimental Animals

Ph. Eur. grade polyethylene glycol 4000 (PEG 4000), stearic acid type 50 (SA), and verapamil–HCl were acquired from Molar Chemicals Ltd. (Halásztelek, Hungary), while all other analytical grade chemicals were from Sigma-Aldrich Ltd. (Budapest, Hungary). Hard gelatine capsules (Coni-Snap, size 00) were gifted to us by Capsugel (Morristown, NJ, USA). Beagle dogs (six, female) were obtained from an authorized breeder (WOBE Ltd., Budapest, Hungary).

### 2.2. Preparation of Solid Foam Capsules

**Figure 1:**
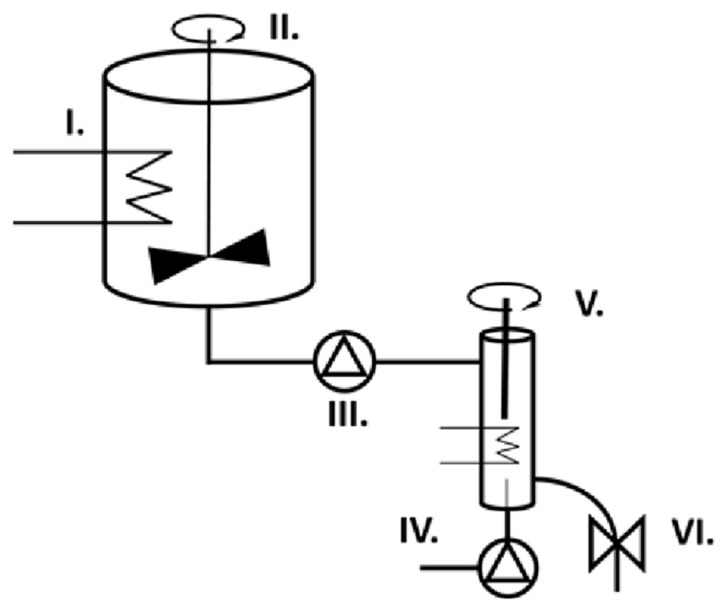
The foaming device contains six main parts. The heated melting vessel (I) and IKA EURO-ST D stirrer (II) are responsible for the homogenous molten suspension that is transferred to the heated foaming cell (IV) by a peristaltic pump (III). The added gas is dispersed by the ULTRA-TURRAX (V) in the melt and dosed by a pinch valve (VI).

Three different compositions of verapamil-containing capsules (Table 1) were produced by a continuous foaming process and a device (Figure 1) that was designed and published earlier by our group [24].

For manufacturing, the following parameters and method were used: PEG 4000 and SA were placed and heated in a melting container at 61 °C while being gently stirred with an IKA^®^ EURO-ST D stirrer (IKA®-Werke GmbH & Co. KG, Staufen, Germany) at 50 rpm. Then, verapamil was dispersed in the molten mixture. The foaming step was done in cycles. Firstly, the heated foaming cell was filled with the molten suspension at a speed of 0.25 mL/s at 56 °C. A total of 2.0 mL of molten dispersion was pumped into the foaming cell, and the IKA^®^ ULTRA-TURRAX^®^ T-25 digital dispersing instrument dispersed air into the dispersion while pumping 2.0 mL of air slowly through the foaming cell at a rate of 0.25 mL/s. Lastly, the hot foam was dosed into size 00 hard gelatin capsules and cooled to room temperature until solidification.

### 2.3. Determination of the Densities of the Samples

To characterize the densities of the solid formulations, the following test was used: The mass and volume of the empty capsules (00 size) were 118 ± 7 mg and 0.91 mL, respectively, in accordance with the manufacturer’s specifications [28]. The density of each sample was calculated by the following formula after the capsules had been completely filled with foam.

(1) $$\rho_{foam}=\frac{m_{sample}-m_{capsule}}{V_{capsule}}$$

where *V_capsule_* is the volume of the empty capsule body (0.91 mL); *m_sample_* is the mass of filled foam in the entire capsule (body and cap), as measured by an analytical balance; *m_capsule_* is the mass of the empty capsule (body and cap) (118 mg); and *ρ_foam_* is the density of the foam.

### 2.4. SEM Analysis

The solid foams were broken to characterize their structures with a Hitachi Tabletop microscope (TM3030 Plus, Hitachi High-Technologies Corporation, Tokyo, Japan). Samples were crushed into halves and mounted on a fixture with graphite-containing, double-sided adhesive tape. The broken surface of the samples was not coated with gold before the SEM examination. A vacuum and low accelerating voltage of 5 kV were used during the investigation. A chemical element analysis (oxygen or chlorine) was done on the broken surface using a Bruker EDX 70 detector.

### 2.5. Dissolution Test

Three random samples were taken for dissolution tests from every composition (V1-V3, preparation steps are described in Section 2.2). Nine hundred milliliters of hydrochloric acid media was used for the dissolution tests (pH = 1.2 without pepsin) using an Erweka DT 800 dissolution tester (rotating paddle method, 75 rpm and 37 °C). Samples (3 mL) were taken after 5, 15, and 30 min, and 1, 2, 3, 4, 5, 6, 7, 8, and 10 h and filtered through a 0.22 μm PES membrane syringe filter. The verapamil content of the samples was determined with a UV/VIS spectrophotometer (Shimadzu UV 1601, Shimadzu Corp. Kyoto, Japan) at 278 nm after dilution with a pH 1.2 buffer. Floatation was checked visually at regular intervals.

### 2.6. Mathematical Analysis of the Drug Release Profiles

The data from the dissolution tests was graphically fitted and evaluated by zero-order, first-order, and Korsmeyer–Peppas models in MS Excel (Microsoft Corporation, Redmond, WA, USA) (Table 2).

### 2.7. Validation of Production

During the validation process, 3 batches of the V2 composition were produced by the foaming cell at 3 different times, following the parameters of the standard protocol that were described in Section 2.2. The densities, API contents and dissolution profiles of the batches were compared.

The data obtained from the dissolution tests of the validation products were compared by similarity (f2) and difference (f1) factors as a model-independent approach for each sample.

(5) $$f1=\frac{\sum_{j=1}^{n}\left|R_{j}-T_{j}\right|\;\;}{\sum_{j=1}^{n}R_{j}}\times 100$$

where *n* is the sampling number and *R_j_* and *T_j_* are the percentages dissolved from the reference and test products at each time point *j*.

(6) $$f2=50\times \log\left\{\;{\left[1+\left(1/n\right)\overset{n}{\underset{j=1}{\sum }}w_{j}{\left|R_{j}-T_{j}\right|}^{2}\;\right]}^{-0.5}\times 100\;\right\}$$

where *w_j_* is an optional weight factor.

The dissolution efficacies (DE) were also calculated as follows.

(7) $$\mathrm{DE}\;=\frac{\int_{0}^{t}y\times dt}{y_{100}\times t}\times 100\%$$

where *y* is the drug dissolved at time *t* (%).

### 2.8. Microtomography and Size Distribution of Foam Cells

The structure of the solid foam was determined by a noninvasive method using a SkyScan 1272 (Bruker, Billerica, MA, USA) compact desktop micro-CT system. The following parameters were used to scan the capsules placed in the sample holder: image pixel size—5 microns, matrix size—1344 × 2016 (rows × columns), Source Voltage—50 kV, and Source Current—200 µA. Flat Field Correction and Geometrical Correction were used. The cross-sectional images were reconstructed from tomography projection images by the SkyScan NRecon package (Version: 2.0.4.2). Post-alignment, Beam-hardening correction, Ring artefact correction, and Smoothing were done. The output formats were DICOM and BPM.

CTAn software was used in the 2D/3D analyses. Thresholding, ROI shrink-wrap, Reload, and 2D and 3D Analysis plugins were applied based on the density analysis. Air bubbles had gray threshold values of 0–40, and the background was removed by ROI shrink-wrap before the analysis. The 3D visualization was done using CTVox software with color coding.

### 2.9. Texture Analysis

The texture analysis was used to characterize the mechanical properties and structures of the dry and wet foamy formulations. Unwetted control samples were not immersed into the dissolution media and tested at room temperature. At first, three random samples of verapamil-containing foam were placed in the Erweka dissolution tester, and the method described in 2.5 was used. Samples were taken after 1, 3, 5, 7, and 10 h, and excess water was absorbed from their surfaces. The Brookfield CT3 texture analyzer was used in the measurements. TA25/1000, an acrylic cylinder (d: 50.8 mm), compressed the samples at a speed of 0.50 mm/s until reaching the maximum load of 4500 g, and the test probe was fixed for a 5 s hold time at the target pressure. After this, the test probe returned to its starting position. Load values are depicted as a function of time (s) to demonstrate texture changes.

### 2.10. Water Uptake and Matrix Erosion Studies

Initial sample weights (*W_ini_*) were measured before the study. Then, the specimens were put into the dissolution vessels, as described in Section 2.5. Samples were slowly removed from the dissolution media with a plastic net after 1, 3, 5, 7, and 10 h and the wet samples (*W_wet_*) were weighed after removing excess water from the surfaces of samples. Then, the samples were dried at 45 °C (Memmert SFE 550, Memmert GmbH, Schwabach, Germany) until reaching a constant weight. Three samples were tested from the selected composition.

The water uptake % (WU%) was calculated as follows:

(8) $$\%\mathrm{WU}=\frac{W_{wet}-W_{dry}}{W_{wet}}\times 100$$

The remaining % of foam was calculated by the next equation:

(9) $$\%\mathrm{Remaining}=\frac{W_{dry}}{W_{ini}}\times 100$$

where *W_wet_* is the the mass of the wet samples, *W_dry_* is the dried mass of the samples, and *W_ini_* is the initial mass of the samples before testing.

Erosion was followed by microtomography by performing CT scans after 1, 3, 5, 7, and 10 h of dissolution.

### 2.11. Floating Strength Determination

An apparatus was built to measure the buoyancy force of samples based on Simons and Wagner [31]. Briefly, a modified tensiometer measured the weight changes of a net holding the sample (Sigma 700, Attension), and using this data, the buoyancy force was calculated. Measurements were made in 500 mL in a buffer of pH = 1.2 at 37 °C under continuous stirring (50 rpm) to ensure similar conditions to those used in in vitro release studies.

### 2.12. Dissolution Test after Long-Term and Accelerated Storage Conditions

To evaluate the drug release profiles after storage, in accordance with ICH guidelines [18], capsules were stored in airtight glass containers under two different conditions. Ten capsules were placed in a climate chamber (ICH110, Memmert GmbH + Co. KG, Schwabach, Germany) for 3 months under accelerated storage conditions (40 ± 2 °C, 75 ± 5% RH), and another ten capsules were stored in an airtight container and kept at room temperature for two years. After storage, samples were inspected for appearance and in vitro drug release. In vitro drug release was compared with the initial dissolution data from samples by similarity (f2) and difference (f1) factors.

### 2.13. In Vivo Pharmacokinetic Study

The pharmacokinetic study was approved by the University of Debrecen Committee of Animal Welfare and by the National Food Chain Safety Office (approval number: HB/06-ÉLB/1657-4/2019) in accordance with national (Act XXVIII of 1998 on the protection and sparing of animals) and European Union (Directive 2010/63/EU) regulations. Six female dogs (10 ± 0.5 kg) were involved in the experiment. Animals were kept at a temperature of between 15 and 21 °C at a relative humidity of 50 ± 10% and a 12 h–12 h light-dark cycle. For all dogs, free access to tap water and 300 g of pelleted food were provided daily. Acclimatization was allowed for 21 days prior to experiments. The experiments were carried out in two steps with a 7-day wash-out period between them. Twelve hours before each experiment, food was withdrawn, allowing only free access to water. Blood samples were collected by cannulation of the cephalic vein. After sample collection, sterile saline (3 mL) was injected to flush the cannula, and sodium heparin solution was used as an anticoagulant.

First, 50 mg of verapamil dissolved in 10 mL of purified water was administered orally to the animals (*n* = 6). Blood samples (2–3 mL) were collected 0.5, 1, 2, 4, 6, 8 and 24 h after administration. Blood samples were centrifuged immediately (3500 rpm for 10 min), and plasma samples were kept at −80 °C until further analysis. After the blood sample collection at 6 h, food was given to all dogs.

Following at 1 week washout period, 120 mg of verapamil-containing foamed capsules was given orally to all dogs (*n* = 6). The procedure (sampling time, sample amounts, plasma separation, and storage) was the same as described above. Food was also given 6 h after capsule administration.

### 2.14. Gastroscopy

After 2 and 4 h, one animal was randomly selected for study. To ensure that the solid drug carrier was in the stomach, the gastric contents of the animals were examined with a video fiberscope (PENTAX Medical Ultrasound Video Fiberscope, EB19-J10U, HOYA Corporation, Shinjuku-ku, Tokyo, Japan), and short videos were captured. Before the examination, the animals were anesthetized with intramuscular ketamine (10 mg/kg, CP-Ketamin—ketamine hydrochloride 10%; Produlab Pharma BV, Raamsdonksveer, The Netherlands), xylazine (1 mg/kg, CP-Xylazin—xylazine-hydrochloride, 2%; Produlab Pharma BV, Raamsdonksveer, Netherlands), and diazepam (0.2 mg/kg, Seduxen–diazepam, 0.5%; Richter Gedeon Nyrt., Budapest, Hungary). Dogs, according to the project authorization, were not euthanized at the end of the experiment.

### 2.15. Quantitative Determination of Verapamilin Plasma

A liquid–liquid extraction method was used for sample preparation. A phosphate buffer (pH 6.0) was prepared in accordance with the European Pharmacopoeia. Briefly, 6.8 g of sodium dihydrogen phosphate was dissolved in 1 L of purified water. The pH was adjusted with sodium-hydroxide. Dog plasma samples (250 µL) were mixed with 0.5 mL of phosphate buffer (pH 6.0). Then, they were extracted with 1.5 mL of a TBME–ethyl acetate (1:1, *v*:*v*) mixture. The organic phase was separated by centrifugation (4000× *g* for 8 min), collected, and evaporated completely with a nitrogen stream at 40 °C [32]. The residue was solubilized in a 500 µL LC mobile phase and measured by the Thermo Accela +LTQ XL LCMS instrument (Thermo Fisher Scientific, Waltham, MA, USA). Chromatographic separation was achieved by a Kinetex XBC18 column (100 × 2.1 mm, 2.6 µm). The mobile phase for elution comprised methanol + 0.1% formic acid (A) and water + 0.1% formic acid (B). The gradient conditions were 0.00 min 70% B, 2.00 min 0% B, 3.10 min 70% B, and 5.50 min 70% B. The column temperature was maintained at 40 °C with the flow rate set at 0.3 mL/min, and a 10.0 µL sample was injected. The optimal ESI ionization parameters were as follows: heater temperature—200 °C; sheath gas—N_2_; flow rate—10 arbitrary units (arb); aux gas flow rate—5 arb; spray voltage—5 kV; capillary temperature—275 °C; and capillary voltage—23.50 V. Sample measurements were run in positive ion mode (MS). The verapamil [M + H]^+^ ion mass (455 *m*/*z*) was detected in SIM mode. For calibration, verapamil was dissolved in the LC mobile phase. To measure recovery, spiked samples containing 9.6, 19.2, and 38 ng/mL verapamil-HCL, prepared as above for injection, were used.

The results were analyzed by Graphpad Prism (Version 6.1 software, GraphPad Software Inc., San Diego, CA, USA) and the relative bioavailability was calculated with the following Equation (10):

(10) $$f_{r}=\frac{AUC_{A}\times D_{B}}{AUC_{B}\times D_{A}}\times 100$$

where *f_r_* is the relative bioavailability (%); *AUC* is the area under the curve (drug concentration in plasma versus time); and *D* is the dose of drug *B* reference sample and *A* test sample.

### 2.16. Statistical Analysis

The statistical analysis was performed with GraphPad Prism^®^ software (Version 6.01, GraphPad Software Inc.). One-way ANOVA and Tukey’s post hoc test were applied to compare the density of the compositions described in Section 2.3. One-way ANOVA and Dunnett’s post hoc test were used when the densities of 3 different V2 compositions were compared, as described in Section 2.7. Differences were considered significant at *p* < 0.05.

## 3. Results

### 3.1. Density of Compositions

**Figure 2:**
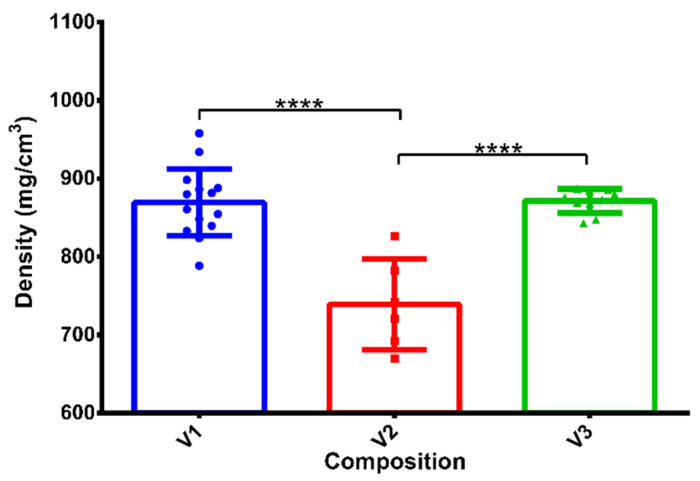
Densities of compositions V1-V3. V2 had the significantly lowest density value. **** indicates statistically significant differences at *p* < 0.0001. The measured values, average values, and standard deviations are presented (*n* = 20).

Three compositions were produced by continuous production, and the final densities were calculated by the equation mentioned above in Section 2.3. The densities of all samples were below 1000 mg/cm^3^, which is necessary to achieve gastric retention. From V1 to V3, the compositions contained increasing amounts of stearic acid as a lipophilic agent. The lowest density was measured for V2. Interestingly, we obtained higher values for V3 despite increasing the lipophilic agent content in the formulation (see Figure 2).

### 3.2. SEM Analysis

**Figure 3:**
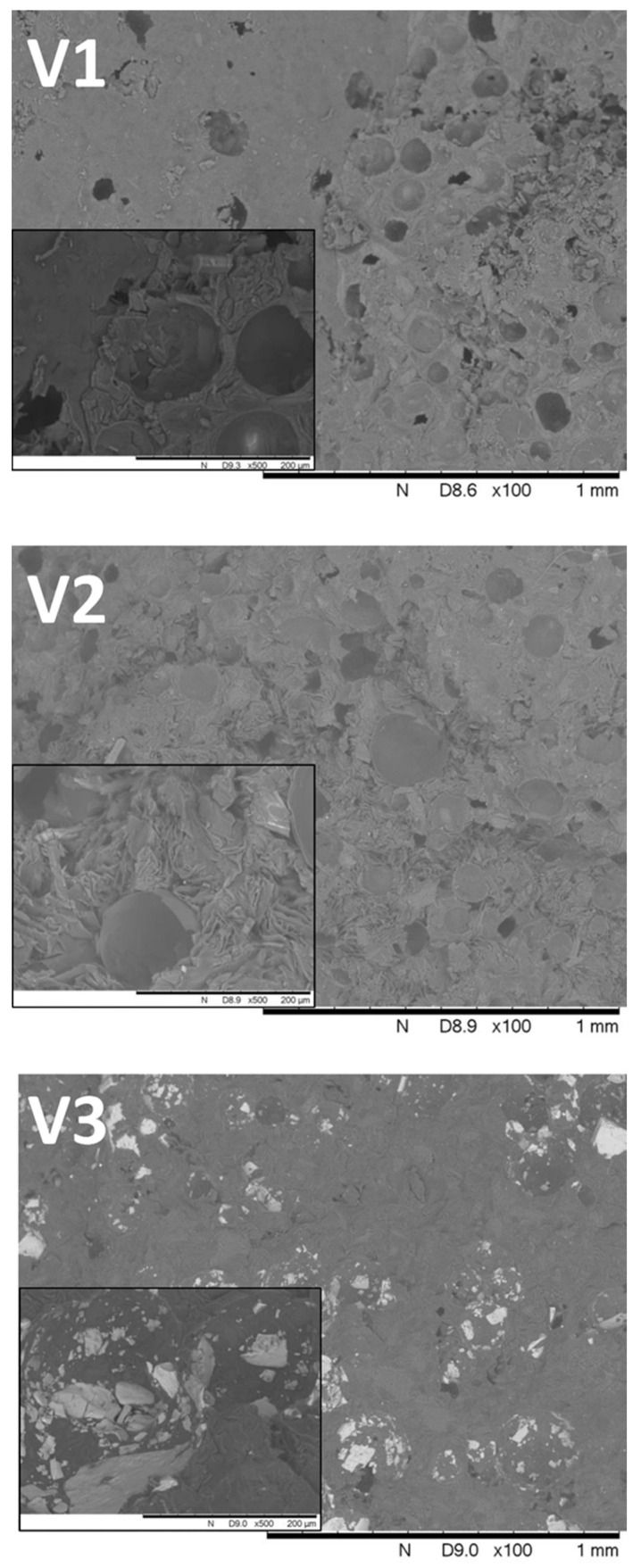
Scanning electron microscopy images of the broken surfaces of V1, V2, and V3. Different locations of the API i can be observed in different matrices. The insets show magnified images of the cavities with verapamil crystals. In V1 and V2, verapamil particles were dispersed through the matrix, while in V3, the white verapamil crystals appeared in the inner surfaces of the cavities. The scale bar of the insets is 200 µm.

The SEM analysis showed the different properties of the compositions (Figure 3). V3 was shown to have a totally different broken surface from V1 and V2. The API accumulated on the surface of the bubbles in general; however, in the case of V3, the white verapamil crystals were found on the inner surfaces of the cavities, while for V1 and V2, the crystals were covered by the matrix on the outer surfaces of the bubbles. In all 3 samples, a high porosity was observed, and this was detected in the form of holes on the fractured surface.

**Figure 4:**
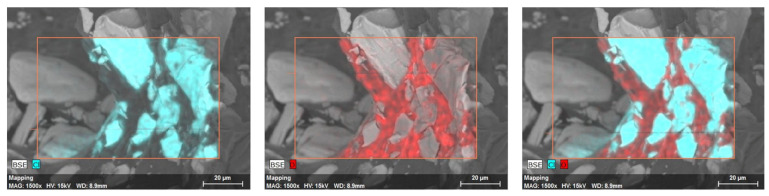
The chemical element analysis of V3 showed the API in solid crystal form (chlorine-containing elements are marked in blue) and separated from the matrix (oxygen-containing elements are marked in red). It accumulated on the inner surfaces of cavities.

The chemical analysis of V3 proved that the API existed on the walls of pores (Figure 4). The oxygen-rich component (red) of the matrix surrounded the verapamil crystals (rich in chlorine, blue).

### 3.3. Dissolution Test

**Figure 5:**
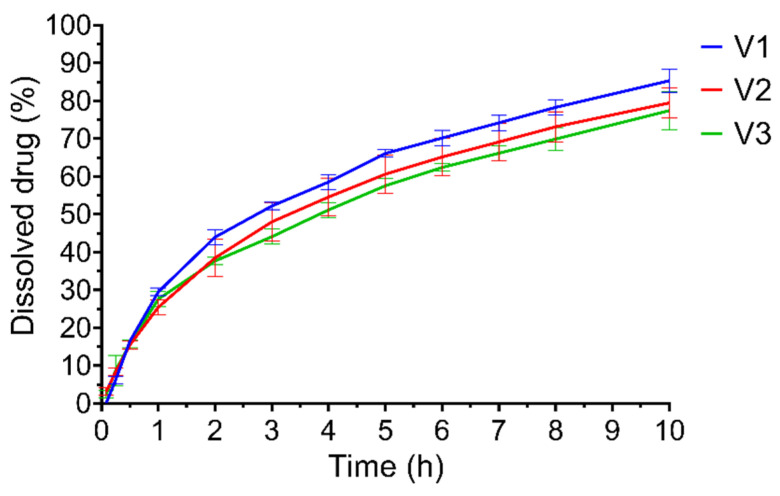
Dissolution profiles of the three compositions. Bars represent mean ± S.D. (*n* = 3).

The dissolution test showed prolonged drug release for up to ten hours in all cases (Figure 5). Flotation was checked visually, and no drugs sunk before the end of the test. The V1 composition reached the highest dissolution rate of 85.3%, while V2 and V3 reached lower values of 79.4% and 77.4%. When the dissolution efficiencies were determined, it was found that V1 showed the fastest release with a value of 70.03%, while for V3, the DE value was only 68.43%. The kinetic profiles are summarized in Table 3. All compositions fitted the best to first-order kinetics pattern; however, V2 fitted best with the target zero-order kinetics (the correlation coefficient was 0.7572). As V2 showed the best properties, this composition was mainly examined in further experiments.

### 3.4. Validation of Production

Validation of the production was then performed with sample V2, and this composition was further analyzed as a final product. Three batches were made using the method described in Section 2.2, and the validation properties of the batches are summarized in Table 4. The average product weight was 890.7 ± 26.7 mg, and no samples deviated more from the average weight than the amount allowed in the pharmacopoeia (Supplementary Materials Figure S1). The average verapamil content was 115.4 mg, and the API content of all samples was in the range of 85–115%. The dissolution profiles of batches were compared with the V2 profile, and none differed from that (Figure S2).

### 3.5. Microtomography

**Figure 6:**
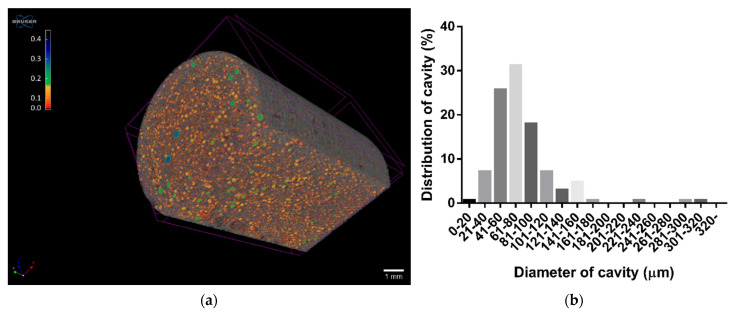
Reconstructed micro-CT image of the V2 composition (**a**) and cavity size distribution (**b**).

The foaming process dispersed air into the molten suspension containing verapamil, and the creation of cavities was confirmed by micro-CT images (Figure 6). The distribution of cavities in the matrix and the size distribution of the bubbles were homogenous. A total of 89.2% of them were in the 20–120 μm range for diameter, and the average diameter of the cavities was around 78 μm (Figure 6b).

**Figure 7:**
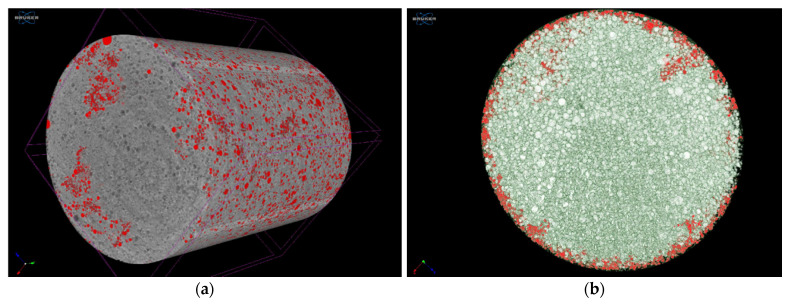
Reconstructed model of the foamed melt presenting the opened cavities in a full capsule (**a**) and cross section (**b**).

The reconstructed model of the foam structure shows a closed spheroid cell structure, but some of the bubbles (bubbles marked with red) can be observed at the edges open to the surface. Some of them form a cavity system in the matrix, although their number is small compared to the whole matrix (Figure 7).

### 3.6. Texture Analysis

**Figure 8:**
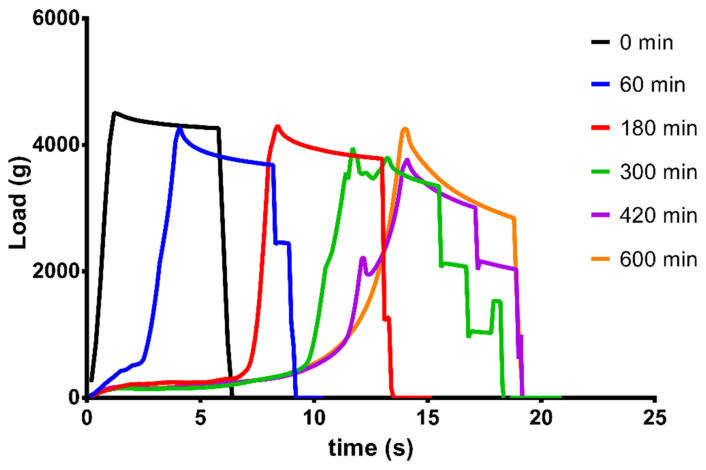
Texture analysis results for the dry, foamed capsule at 25 °C (0 min) and dissolution-coupled texture analysis curves after 60, 180, 300, 420, and 600 min of dissolution at 37 °C. Average values are presented (*n* = 3).

The results of the texture analysis are presented in Figure 8. Despite the high porosity, a hard structure is presented. Using a compression load of 4500 g on the foam, no cracks, fractures, or other injuries were detected on the dry sample at 25 °C. During the drug release, the initial hardness of the samples decreased. After 60 and 180 min of dissolution, a soft layer was found around the hard core, which was easily removable, and the two parts easily separated by micro-CT scans, as shown in Figure S3. At 300 and 420 min of dissolution, some cracking was seen on the curves of samples. The hard core became fragile, and the core was broken from 3810 g (for the 300 min sample) and 3770 g (for the 420 min sample). Thorough wetting was detected after 600 min, and the breakable hard core disappeared.

### 3.7. Water Uptake and Matrix Erosion Studies

**Figure 9:**
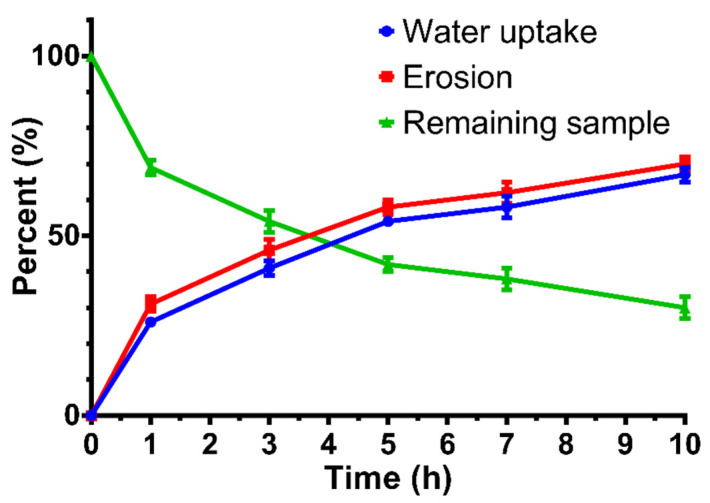
Water uptake, erosion, and remaining sample (%) vs. time (h) curve of the V2 compositions. Bars represent mean ± S.D. (*n* = 3).

During dissolution, the mass of the matrix decreased continuously, as shown in Figure S3. A summary of the erosion and water uptake data is shown in Figure 9. The matrix did not show swelling, and the sum of remaining matrix and the water content was constant. Only 30% of the sample remained after the test.

### 3.8. Floating Strength Determination

**Figure 10:**
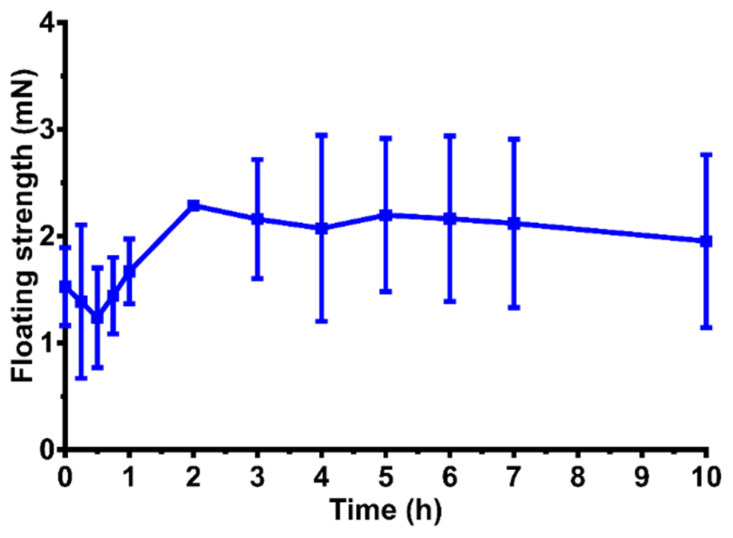
Floating behavior of formulation V2 in a buffer of pH 1.2 at 37 ± 0.5 °C. The mean ± S.D. are presented (*n* = 3).

At the zero-time point, the sample was able to float on top of the acidic media and generate a buoyancy force of 1.5 mN. During the swelling and erosion of the capsule shell, the buoyancy decreased to 1.2 mN. Then, it started to increase and entered a plateau phase at around 2 mN until the API was completely released (Figure 10).

### 3.9. Dissolution Test after Long-Term and Accelerated Storage Conditions

Three parallel dissolution studies were performed to prove appropriate drug liberation after 3 months and 2 years, respectively. The dissolution profiles of the samples were compared by similarity and difference factors (Figure S4 and Table S1). The difference factor was less than 5.00 in all cases, and the similarity factor was greater than 50.00.

### 3.10. In Vivo Study

**Figure 11:**
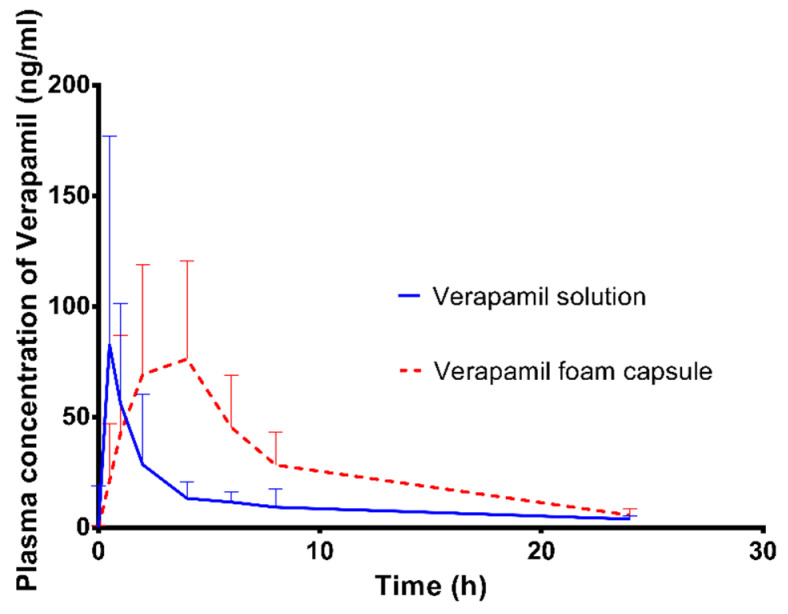
Mean plasma Verapamil–HCI concentrations and standard deviations after a single oral dose of the reference solution and foam capsule were administered to beagle dogs (*n* = 6).

The solutions containing verapamil–HCl (50 mg) and the solid gastroretentive foam were orally administered to the animals in the bioavailability test. The verapamil plasma concentrations were compared and are shown in Figure 11.

**Figure 12:**
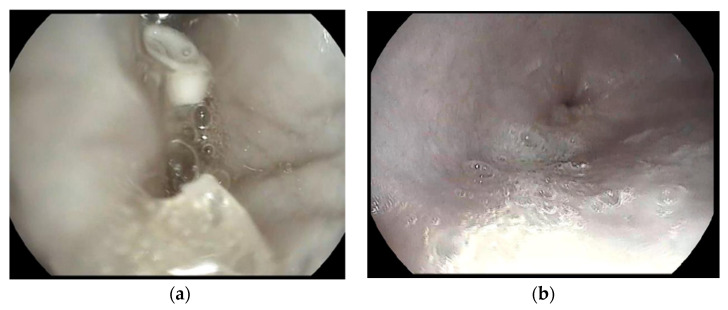
A gastroscopy scan 2 h after administration proved the gastroretention of the foamed capsule. The white body of the residue of the formulation can be seen on the upper part of the image (**a**). The capsule was eliminated from the empty stomach 4 h after administration (**b**).

Gastroscopy was performed, and gastric retention after two hours was proven (Figure 12a). After 4 h, the capsule was eliminated from the stomach (Figure 12b).

The main pharmacokinetic properties are presented in Table 5. The capsule reached the maximum concentration in plasma 4 h after administration, while the solution reached this level earlier, after 0.5 h. In the case of the foam, the plasma concentration was above 50 ng/mL for 5 times longer than in the case of the solution, while the initial concentration was just 2.5 times higher. The relative bioavailability was 99.3%.

## 4. Discussion

In the present study, our aim was to increase the bioavailability of verapamil–HCl by applying a new solid foam formulation based on melt foaming. Verapamil formulations must be administered frequently, but this can be reduced by the use of GR formulations [8,9,22]. From a technological point of view, the thermal stability of verapamil is also favorable for the formulation using technology developed by our research team previously [33]. During the production of verapamil solid foam, the foam cell temperature was higher by 2 °C than that previously described with the BaSO_4_ composition due to the higher melting point of the verapamil mixture [3,24]. Three compositions were produced with 15% verapamil–HCl, containing 80–120 mg of verapamil per capsule, which corresponds to the active substance content available in the literature and to the marketed preparations [20,34,35]. An increase in the stearic acid content from 10% (V1) to 12.5% (V2) helped to form the foam structure, as described previously by Vasvári et al. [3], but it is important to mention that the excessive stearic acid content reduced foaming. For V3, the increased density could be the reason for the changes in the wetting of the API particles by the molten matrix [36,37,38]. The SEM images support the altered solid particle location. In the case of V3, verapamil crystals were found in the inner surface of the cavity, while in the cases of V1 and V2, they were dispersed in the matrix and localization in the cavities was not specific. The foaming efficacy does not depend on only the hydrophilicity of the matrix, and according to our experiences, the particle size of the solid phase also significantly affects the degree of foaming. Previously, we successfully produced a lower-density product using smaller drug particles (314 nm ± 115) of BaSO4 with the abovementioned technology [24], and the use of verapamil particles with an average particle size (13.4 µm ± 11.2) resulted in a higher density. The drug dissolution test showed prolonged drug release in all compositions. Compared with marketed preparations, the drug release was slower than from Isoptin SR but showed similar kinetics to Calaptin SR [22,39]. During validation, three parallel productions were performed and compared, and the validation results comply with FDA and GMP regulations [40]. The micro-CT scans showed a high porosity, a homogenous distribution of bubbles in the matrix, and a monodispersed cavity size distribution. In general, open pores were shown to accelerate the rate of dissolution. Our capsule has few open pores, so their presence does not contribute significantly to the erosion of the preparation [41]. The texture analysis during the dissolution showed that the samples remained hard until 300 min. At the end of the dissolution process, 30% of the initial weight was still present, and the matrix became plastic and was easily removed by the grinding or churning motions of the stomach [2,10]. The PEG and API dissolved from the matrix, while the SA remained undissolved during the dissolution test. The composition did not show the ability to swell. In a dissolution study performed after storage under accelerated conditions, the formulation was compared to the initial formulation with factors f1 and f2, and the results met expectations. A similar result was observed for capsules stored under extended 2-year standard conditions. The results of the dissolution studies showed that no major changes occurred in the verapamil-containing solid foam matrices which influence the drug dissolution. The in vivo pharmacokinetics study proved that the GR system is able to significantly change the pharmacokinetic parameters of verapamil. For the verapamil solution, the in vivo plasma concentration of verapamil was correlated with the published conventional verapamil–HCl pill, Staveran [9]. In this study, verapamil solution was used to reveal the absorption properties and pharmacokinetics of pure verapamil from the GI tract and to avoid the influence of excipients or the liberation of verapamil from a formulation. By comparing the results of the solid foam capsule with a retard pill, Isoptin SR, the t_max_ shift was observed. Instead of a sharp peak, a plateau was observed, and plasma levels could be further maintained in the therapeutic range [22], reaching a relative bioavailability of 99.3%. The verapamil plasma levels correlated well with the residency of the capsule in the stomach, as shown by the gastroscopy images. Even if the stomach of the animal was empty and showed fast motility, the capsule could be detected 2 h after administration. The dissolution results of verapamil foams are reflected in the in vivo data. The in vitro–in vivo correlation is shown in Figure S5, and in vivo absorption was predicted by the in vitro tests. With the application of high porosity verapamil foam, the gastroretentive purposes of controlled release and stomach retention were achieved simultaneously [42]. In terms of our results, it can be said that our preparation is suitable for gastroretention and can reduce the frequency of administration of the preparation, thus achieving better adherence. Our results were confirmed by both in vitro and in vivo experiments.

## 5. Conclusions

In our current study we produced verapamil-loaded gastroretentive capsules by continuous production using a foaming device for the first time. After optimizing the apparent density of the product by considering the SA contents, production runs were validated. Micro-CT scans revealed a closed cell structure where the main fraction of the voids was smaller than 120 microns. The texture analysis results confirmed a hard structure, even after 5 h of dissolution. Despite continuous erosion of the PEG matrix, floating strengths of the samples remained stable during dissolution. Our studies confirm that the first-order drug release was preserved, even after 2 years of storage. The in vivo pharmacokinetic study verified the prolonged release of the API from the matrix. The maximum drug plasma concentration was reached after 4 h of administration. Compared to the verapamil oral solution, a relative bioavailability of 99.3% was reached. Summarizing our results, we can state that our foaming device could be used successfully to produce low-density molded capsules with in vivo gastroretentive properties by continuous operation.

## Figures and Tables

**Table 1:** The composition of the capsules prepared by the continuous foaming process.

Composition ^1^	PEG 4000	Stearic Acid	Verapamil-HCl
V1	75.0%	10.0%	15.0%
V2	72.5%	12.5%	15.0%
V3	70.0%	15.0%	15.0%

^1^ For each composition, a 120.0 g molten dispersion was used for production. (% is the abbreviation for m/m %.).

**Table 2:** Mathematical models of drug release profiles.

Model	Equations [29,30]		Graph
Zero-order	$Q_{t}=Q_{0}+k_{0}t$	(2)	The graph of the drug-dissolved fraction vs. time is linear.
First-order	$Q_{t}=Q_{0}\times e^{-k_{1}t}$	(3)	Linear graph of the released amount of drug (expressed by decimal logarithm) vs. time.
Korsmeyer-Peppas model	$\frac{Q_{t}}{Q_{\infty }}=k_{kp}t^{n}\left(\mathrm{up}\;\mathrm{to}\;\frac{Q_{t}}{Q_{\infty }}\geq 0.6\right)$	(4)	Straight line graph of released drug vs. the square root of time.

where *Q*—amount of drug released at time *t*; *Q_0_*—the initial amount of drug at time 0; *Q_t_*—the amount of drug remaining at time *t*; *Q_t_/Q_∞_*—fraction of drug released at time *t*; *k_0_, k_1_,* and *k_kp_*—kinetic constants for zero-order, first-order, and Korsmeyer–Peppas models, respectively; *n*—release exponent, indicative of the drug release mechanism.

**Table 3:** Model fitting results for the dissolution data of the V1, V2 and V3 composition.

	Correlation Coefficient (R^2^)		
Composition	Zero ^1^	First ^1^	Korsmeyer–Peppas ^1^
V1	0.7467	0.9740	0.9371
V2	0.7572	0.9646	0.9589
V3	0.7511	0.9881	0.9619

^1^ Values were obtained by graphical analysis.

**Table 4:** Validation properties of V2 compositions. Three batches were produced at different times.

	V2 Composition ^1^		
Validation Properties	Batch I	Batch II	Batch III
Average weight of batch ± SD (mg)	885.9 ± 24.8	898.8 ± 36.5	894.5 ± 18.15
Maximum absolute deviation from average weight (all batches %) ^3^	5.0	8.8	5.5
Average API content of batch ± SD (mg)	106.9 ± 4.0	117.2 ± 8.5	122.1 ± 5.2
Maximum absolute deviation from average API content of all batches (%) ^3^	11.9	10.7	11.4
Difference factor ^2^	4.19	4.95	2.60
Similarity factor ^2^	99.98	99.98	99.99

^1^ an average of at least 20 samples; ^2^ compared to the preformulation of V2; ^3^ compared to the average values of all batches.

**Table 5:** The main pharmacokinetic properties are summarized. The Verapamil foam capsule showed significantly different results in T_max_ and AUC compared to the solution.

Composition	Verapamil Solution	Verapamil Foam Capsule	Analysis of Variance ^1^
C_max_ (ng/mL)	90.24 ± 89.02	101.20 ± 46.15	NS
T_max_ (h)	1.00 ± 0.54	2.67 ± 1.51	IS
AUC (0–24 h)	254.5 ± 175.4	682.4 ± 297.1	IS

^1^ IS indicates a statistically significant difference at *p* < 0.05, while NS indicates no statistically significant difference.

## Data Availability

Not applicable.

## Supplementary Materials

The following supporting information can be downloaded from https://www.mdpi.com/article/10.3390/pharmaceutics14020350/s1. Figure S1: Weight validation of the V2 product. The measured weight, average weight, and standard deviations are presented; Figure S2: The dissolution profiles of the batches and V2 preformulation; Figure S3: Micro-CT scans of matrix erosion during dissolution; Figure S4: Three parallel dissolution studies of fresh samples after 3 months in a climate chamber and after 2 years at room temperature. Figure S5: In vitro–in vivo correlation of formulation V2; Table S1: The dissolution profile of the stability test was compared by similarity and difference factors.
