# Supplementary material for: In Vitro and In Vivo Studies of a Verapamil-Containing Gastroretentive Solid Foam Capsule
Source: Pharmaceutics. 2022 Feb 2;14(2):350. doi: 10.3390/pharmaceutics14020350 (PMC8878168; doi:10.3390/pharmaceutics14020350)
Supplement: Supplementary file 1 [file pharmaceutics-14-00350-s001.zip › pharmaceutics-1536882-supplementary.pdf]

pharmaceutics

Supplementary Materials: In Vitro and In Vivo Studies of a Verapamil-Containing Gastroretentive Solid Foam Capsule

Ádám Haimhoffer, Gábor Vasvári, István Budai, Monika Béresová, Ádám Deák, Norbert Németh, Judit Váradi, Dávid Sinka, Ildikó Bácskay, Ferenc Fenyvesi

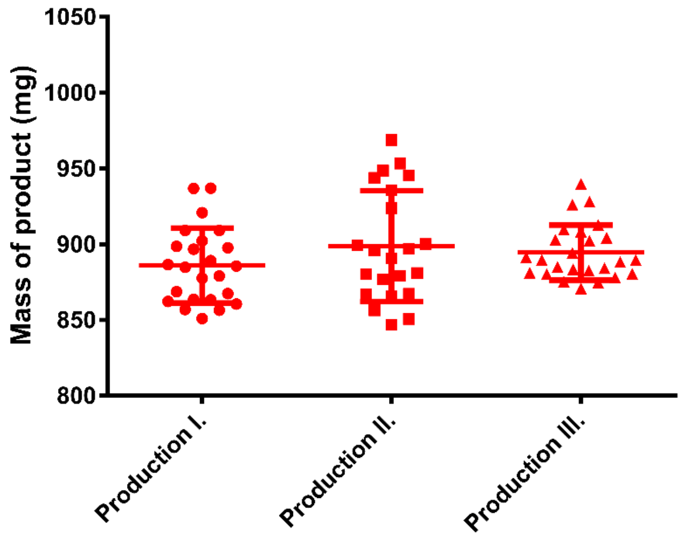

Figure S1. Weight validation of V2 product. Data present the measured weight, average weig, and standard deviations.

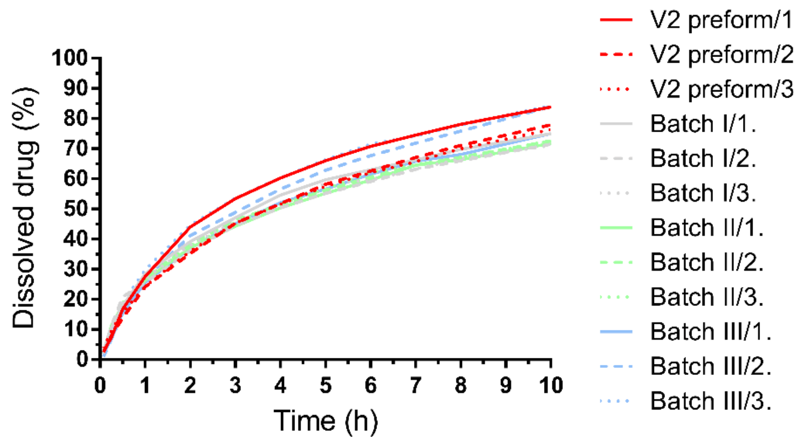

Figure S2. The dissolution profiles of batches and V2 preformulation.

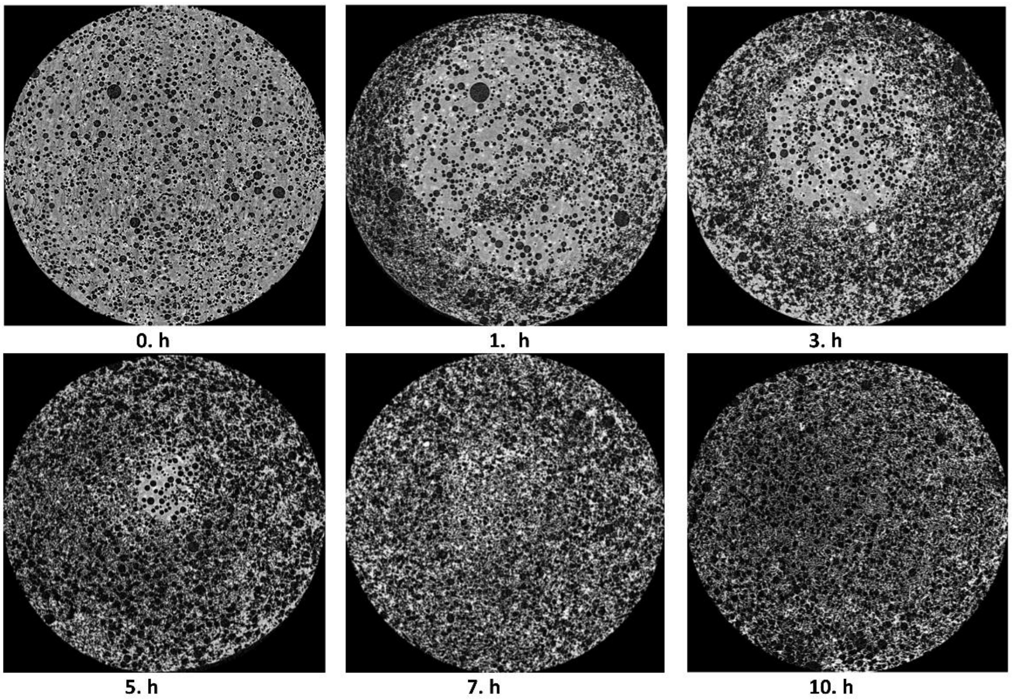

Figure S3. Micro-CT scans of matrix erosion during dissolution.

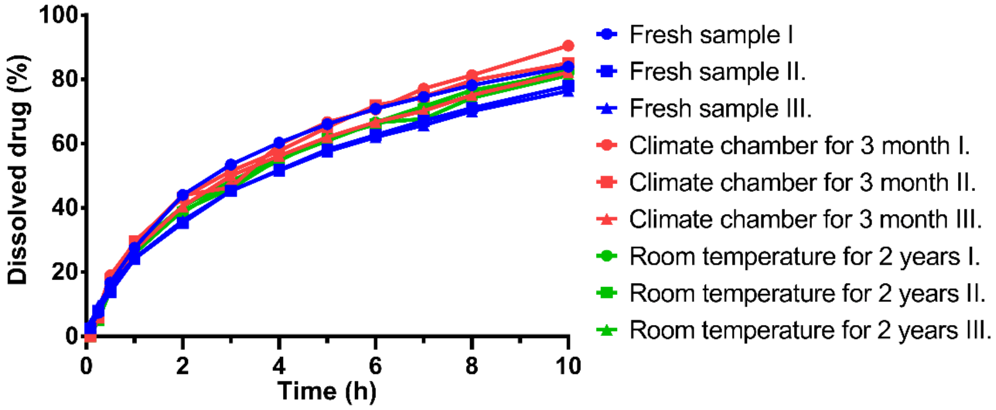

Figure S4. Three parallel dissolution studies of fresh sample, after 3 months in climate chamber and after 2 years at room temperature.

Table S1. The dissolution profile of stability test was compared by similarity and difference factors.

Validation Properties	3 Months	2 Years
Difference factor[1]	6.27	1.80
Similarity factor[1]	61.80	71.74

[1] compare to the fresh sample

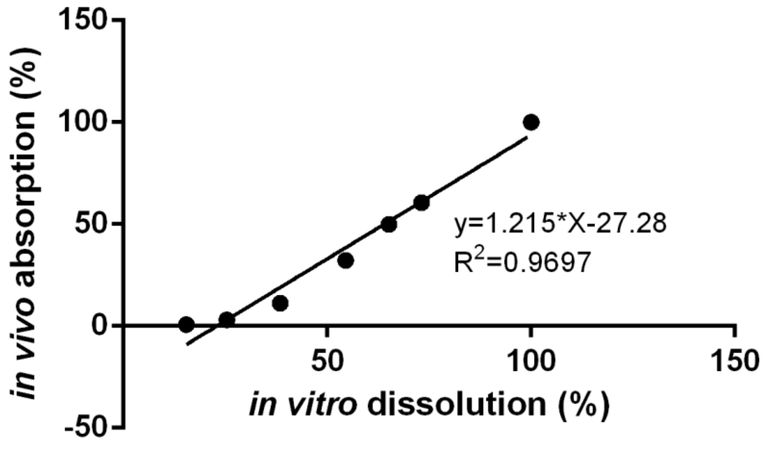

Figure S5. In vitro–in vivo correlation of formulation V2.
